# Retraction: Melanin pigment of *Streptomyces puniceus* RHPR9 exhibits antibacterial, antioxidant and anticancer activities

**DOI:** 10.1371/journal.pone.0272186

**Published:** 2022-08-03

**Authors:** The PLOS ONE Editors

The *PLOS ONE* Editors retract this article [1] because it was identified as one of a series of submissions for which we have concerns about authorship, competing interests, and peer review. We regret that the issues were not addressed prior to the article’s publication.

BH and SB either did not respond directly or could not be reached. AA, AHA, FB, HAEE, MM, RP, RZS, KR, and SH did not agree with the retraction.
